# Dynamic Obstacle Avoidance Using Bayesian Occupancy Filter and Approximate Inference

**DOI:** 10.3390/s130302929

**Published:** 2013-03-01

**Authors:** Ángel Llamazares, Vladimir Ivan, Eduardo Molinos, Manuel Ocaña, Sethu Vijayakumar

**Affiliations:** 1 Department of Electronics, University of Alcalá, 28871 Alcalá de Henares, Madrid, Spain; E-Mails: eduardo.molinos@depeca.uah.es (E.M.); mocana@depeca.uah.es (M.O.); 2 Institute of Perception, Action and Behaviour, University of Edinburgh, Edinburgh EH8 9AB, UK; E-Mails: v.ivan@ed.ac.uk (V.V.); sethu.vijayakumar@ed.ac.uk (S.V.)

**Keywords:** autonomous navigation, obstacle avoidance, optimal control

## Abstract

The goal of this paper is to solve the problem of dynamic obstacle avoidance for a mobile platform by using the stochastic optimal control framework to compute paths that are optimal in terms of safety and energy efficiency under constraints. We propose a three-dimensional extension of the Bayesian Occupancy Filter (BOF) (Coué *et al. Int. J. Rob. Res.* **2006**, *25*, 19–30) to deal with the noise in the sensor data, improving the perception stage. We reduce the computational cost of the perception stage by estimating the velocity of each obstacle using optical flow tracking and blob filtering. While several obstacle avoidance systems have been presented in the literature addressing safety and optimality of the robot motion separately, we have applied the approximate inference framework to this problem to combine multiple goals, constraints and priors in a structured way. It is important to remark that the problem involves obstacles that can be moving, therefore classical techniques based on reactive control are not optimal from the point of view of energy consumption. Some experimental results, including comparisons against classical algorithms that highlight the advantages are presented.

## 1. Introduction

Autonomous vehicles have nowadays become popular for applications such as surveillance and passenger transport. In both cases the safety and efficiency of these systems depends on the ability of the autonomous navigation system to deal with unpredictable dynamic changes in the environment. Autonomous navigation systems have been studied extensively in the literature [1,2]—core fundamental requirements for such systems include perception, localisation, planning and actuation. On-board sensors are used to perceive the state of the environment while the localisation stage is usually based on fusion of sensory information and a priori map to determine the location of the vehicle within the global frame. Once the vehicle's location has been determined, the sequence of actions necessary to reach the goal can be computed within the planning stage. The resulting plan has to satisfy various constraints such as: holonomic constraints, safety, traffic rules, energy efficiency etc. Finally, the actuation stage is responsible for executing the plan.

The localisation stage can be greatly improved by using Global Positioning System (GPS) or its enhanced version: the Differential GPS (DGPS) [3]. This provides a global reference for vehicle with the accuracy of a few centimetres in the case of DGPS. However, when a GPS receiver is deployed in urban environments with high buildings or underground tunnels, the signal can suffer multipath fading or even Line-Of-Sight (LOS) blockage which renders this sensor inoperative.

For autonomous vehicles to achieve safe operation, understanding safe operation as an obstacle avoidance task that preserves the integrity of the robot and the other objects or people in a dynamic environment, a combination of a priory map with sensory information that comes from sensor fusion [4,5] is required. The vehicle can be then safely guided through a mesh of connected way points. Such maps are usually obtained in a semi-autonomous way [6] or using Simultaneous Localization and Mapping (SLAM) techniques [4,7,8] to reduce the uncertainty of localisation and mapping processes by doing both at the same time.

In addition to localisation, a robust autonomous mobile platform requires an obstacle avoidance system. Such system ensures that a vehicle navigates safely around the obstacles while trying to reach its goal. Obstacle avoidance can be divided into global and local approaches. While the former approach assumes a complete model of the environment, such as the potential field methods, the local methods require only partial observability of the environment at the cost of guaranteeing only local optimality. However, computational cost is much lower for local methods and they can be often implemented in the form of reactive controllers. These reactive methods take control of the robot when an obstacle is detected to prevent collision. They use the nearest portion of the environment modelled using the current sensor observation. Some representative examples are Vector Field Histogram (VFH+) [9], Nearness Diagram (ND) [10], Curvature-Velocity Method (CVM) [11], its improved version the Lane-Curvature Method (LCM) [12] and the Beam-Curvature Method (BCM) [13].

One of the major drawbacks of the reactive methods is that they do not take into account the dynamic changes of the environment and assume that all obstacles are static. Therefore, they can not predict their motion. This is, however, an unrealistic assumption especially when the vehicle deals with a high uncertainty over the position, shape and velocity of the obstacles and it is still a challenge for real world applications [14,15]. It is also crucial to combine the available sensory input in a structured manner. In [16] the authors propose to formulate the planning problem as inference on a graphical model. This framework allows to combine sensory information, constraints and multiple goals in the form of task variables that may be represented in different spaces such as configuration space, end-effector space (typical for reaching tasks) or even abstract spaces such as topology based representations of the environment. Hierarchical task variables can be constructed to provide both low level control at the level of dynamics and high level control at the level of task objectives.

This paper, which extends our previous work recently sketched in [17], proposes an obstacle avoidance system that takes into account not only the safety of our vehicle around the dynamic obstacles but also optimality of the motion in terms of additional constraints such as the energy consumption. We demonstrate how dynamic obstacles are treated within our navigation system, and how the planning stage can be improved by solving the problem within a stochastic optimal control framework. We reduce the energy consumption of our vehicle by, firstly, using a new probabilistic model of the environment within the perception stage, and secondly by optimising the trajectory using the Approximate Inference Control (AICO). We compare our proposed method with the classical obstacle avoidance algorithms (VFH+, CVM, LCM and BCM).

The rest of the paper is organized as follows: Section 2 shows the proposed method; Section 3 describes the experiments and the actual results; and finally, in Section 4 we conclude and discuss the future works.

## 2. Proposed Method

In this section we propose an extension of the method for avoiding dynamic obstacles in three dimensions while minimising the energy consumption. To tackle this problem, we obtain a probabilistic model of the dynamic environment, then we use this model to predict the motion of the obstacles inside our perception stage and finally, we use optimal path planning using approximate inference to improve the planning stage based on an energy consumption model.

### 2.1. Probabilistic Model of the Dynamic Environment

**Figure 1.**
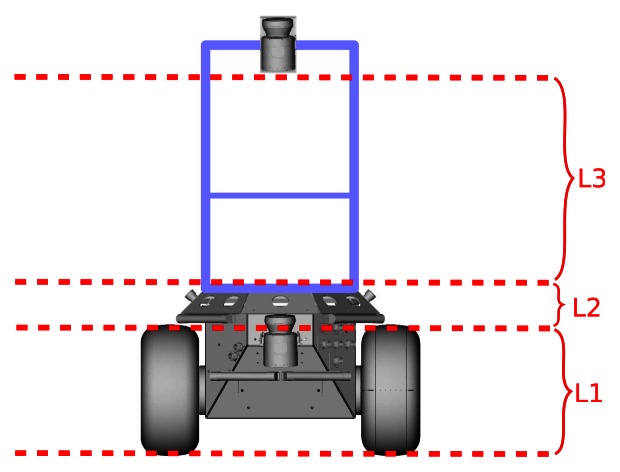
Discretisation levels.

We use the Bayesian Occupancy Filter (BOF) [14] to compute a robust estimate of the position of the obstacles. BOF has been successfully used to detect obstacles in a flat world, but does not tackle for obstacle detection in 3D. In order to add the *height* into the BOF, we use the information provided by our laser range sensor, mounted at an angle with respect the ground plane, as stated in our previous work [18]. The height has been discretised at 3 levels, *L*1 (the wheel height), *L*2 (the mid-body height) and *L*3 (the overhead height), converting the cells into cubes. In order to compute the three-dimensional BOF, all the 3D points inside each cube are projected to the corresponding cell and level. Figure 1 shows the discretisation at three levels with respect to the robot.

The Bayesian Occupancy Filter can be implemented as a loop of a prediction and estimation steps. The authors of [14] suggest that prediction (Equation (1)) and the estimation (Equation (2)) steps can be computed as follows:

(1) $$P(e_{n}^{t}|n^{t},u^{t-1})\propto \sum_{n^{t-1}}P(n^{t}|n^{t-1},u^{t-1})P(e_{n}^{t-1}|n^{t-1})$$

(2) $$P(e_{n}^{t}|z^{t},n^{t})\propto \sum_{m=1}^{S}(\prod_{s=1}^{S}P(z_{s}^{t}|e_{n}^{t},m))$$

where $e_{n}^{t}$ is the occupancy of the cell *n* at time *t*, *u* is the command issued at time *t* — 1, *z* are the observations and *m* is matching between a cell and an observation. *P*(*n^t^*∣*n^t^*^−1^, *u^t^*^−1^) is then the transition probability defined by the vehicle dynamics. The standard BOF framework has several issues with the velocity estimation. Firstly, this framework assumes that the velocity of each grid cell is constant [19]. Secondly, the discretisation has to be performed also in the velocity space, meaning that a separate estimate for each pair of velocities (*v_x_*, *v_y_*) is required. This discretisation for a large range of possible velocities together with calculations of static objects result in high computational costs. For these reasons, other authors proposed object detection and clustering techniques to obtain the objects' velocities [20] with an additional constraint that the position of the obstacle has to remain within a bounded neighbourhood.

**Figure 2.**
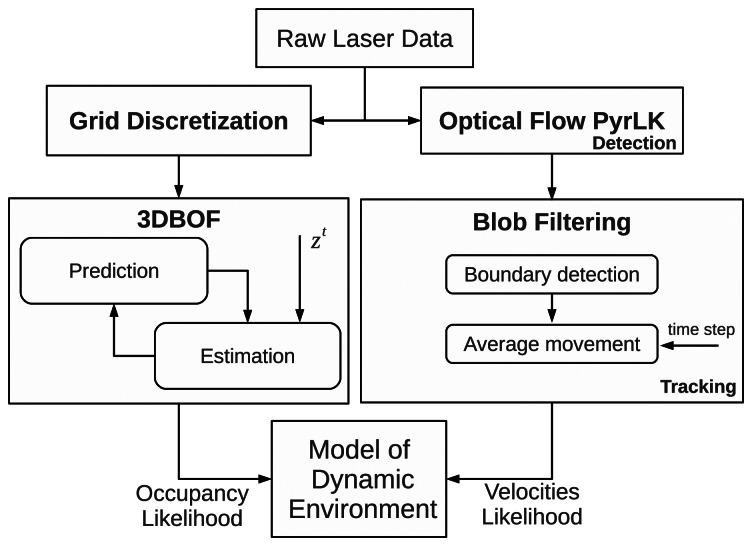
Probabilistic model of the dynamic environment.

We have improved the system in terms of efficiency, by adding a stage to detect the relative velocities of the obstacles without assuming constant velocity objects or discretised velocities. Figure 2 shows the flow diagram of our proposed model.

Firstly, we capture a frame using the laser data. This frame has the form of a zenith image of the detected obstacles. We then estimate the movement of the obstacles between two frames by computing the pyramidal implementation of Lukas Kanade optical flow algorithm [21]. Then, we introduce a blob filtering stage in two steps: (1) we detect the boundaries of the objects and (2) we obtain the average motion of all the cells inside each boundary. Each cell has to exceed certain occupancy threshold in order to reduce the noise in the output of optical flow and the computational cost. This average value and the time step are used to compute the relative velocities between the obstacles with respect to the robot's local frame of reference (along local axis *x* and *y*), while the velocity along the *z* axis is not taken into account due to the assumption that the robot moves on a plane. The result of the perception stage is a dynamic occupancy grid providing an estimation of velocity and the occupation probability.

**Figure 3.**
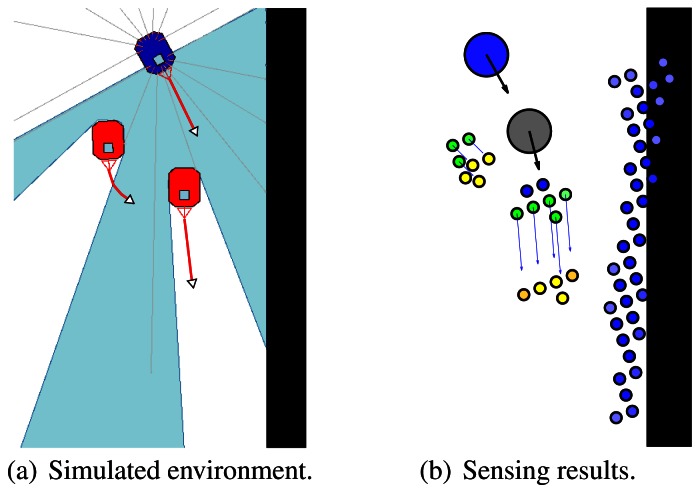
Simulated environment and sensing results.

The output of our probabilistic model is illustrated in Figure 3. Left image shows the simulated environment. Our robot is represented in blue colour and the moving obstacles are red. Right image shows the sensing results. The blue circle is the robot at the current time step, the grey circle shows the predicted future position of the robot, moving obstacles at current time step are represented with green dots, the predicted future positions of moving obstacles are shown as yellow dots and static obstacles are shown as blue dots. The occupancy probability value is given by the darkness value of each dot.

### 2.2. Energy Consumption Model

In order to compare our method to the classical obstacle avoidance algorithms in terms of energy, we only take into account the power demand of the robot's motors. We assume that the rest of the equipment has constant energy consumption and therefore, it cannot be improved any further. We also assume that the power demands of the robot's motors are based on overcoming inertia, road grade, tyre friction, and aerodynamic loss. This road-load methodology was mainly introduced by [22]. The power demand (in Watts) is the tractive power as denned by Equation (3):

(3) $$P=mv[a(1+ɛ)+gR_{G}+gK_{R}]+\frac{1}{2}\rho K_{D}A_{F}v^{3}$$

where *m* is vehicle mass in metric tones (0.077 in our case), *v* is vehicle speed (assuming no headwind) in m/s, *a* is vehicle acceleration in m/s^2^, *ε* is a mass factor accounting for the rotational masses, is assumed to be 0.1 [23], *g* is acceleration due to gravity (9.8 m/s^2^), *R_G_* is road grade (0.0 in our case), *K_R_* is rolling resistance - this value for radial tires can range from 0.008 to 0.013 for a majority of the on-road passenger car tires but can be larger depending on tire pressure, temperature, ground surface, and speed [24,25] (a medium value in the range ≈ 0.009 is assumed [22]), *ρ* is air density (≈ 1.2 kg/m^3^), *K_D_* is aerodynamic drag coefficient (≈ 0.3 [22]) and *A_F_* is the frontal area in meters^2^ (≈ 1 m^2^ in our case).

The robot speed that we use to obtain the power demand is provided by the kinematic model of the robot based on the angular speed of the wheels for each time step (100 ms in our case). According to this, we assume that the robot is moving with linear speed between execution steps. On the other hand, our planning and almost all the classical algorithms do not allow the robot to describe sharp turns or spin.

### 2.3. Optimal Path Planning Using Approximate Inference

**Figure 4.**
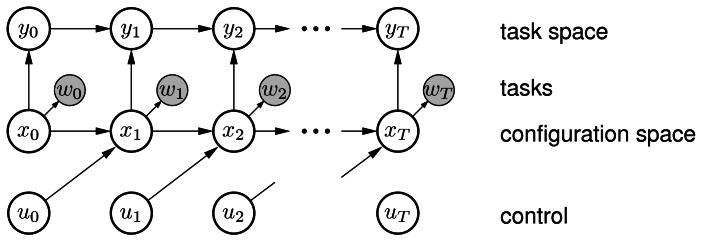
Graphical model of AICO in configuration and task space.

The stochastic optimal control has been successfully used to solve optimisation problems in robotics [26-28]. We have decided to formulate the path optimisation problem within the Approximate Inference Control (AICO) framework [16,29]. The state of the robot is defined by *x_t_* = (*r_x_*, *r_y_*)—the position of the robot on the ground plane (*r_x_*, *r_y_*) and its derivative in the dynamic case. The transition probability is defined by a linear control process with Gaussian noise:

(4) $$P(x_{t+1}|x_{t},u_{t})=N(x_{t+1}|A_{t}x_{t}+a_{t},Q_{t}+B_{t}H^{-1}B_{t}^{T})$$

given state *x_t_* where *A_t_*, *a_t_*, *B_t_* define the linear system that approximates the state transition, *Q_t_* is the covariance of the system noise and *H_t_* is the covariance of the uniform prior over *u_t_*. The control *u_t_* has been integrated out to simplify the equation (refer to [16]). Our goal is to compute a path that minimizes the total expected cost from time *t_0_* to the final time *t_T_*:

(5) $$C(x_{0:T},u_{0:T})=\sum_{t=0}^{T}c_{x}(x_{t})+c_{u}(u_{t})$$

where *c_x_*(*x_t_*) is the state dependent cost (defined by sum of angular velocity and reciprocal distance to static and dynamic obstacles) and *c_u_*(*u_t_*) is the control cost (defined by Equation (3)). The problem can now be described by a graphical model:

(6) $$p(x_{0:T},u_{0:T})\propto P(x_{0})\prod_{t=0}^{T}P(u_{t})\prod_{t=1}^{T}P(x_{t}|u_{t-1},x_{t-1})\prod_{t=0}^{T}P(w_{t}=1|x_{t})$$

where *P*(*u_t_*) is the control prior reflecting the control cost and *P*(*w_t_* = 1∣*x_t_*) is the probability of receiving low cost reflected by constantly observing a random variable *w_t_* = 1. The binary random variable has the conditional probability *P*(*w_t_* = 1∣*x_t_*, *u_t_*) = exp(*C*(*x*_0:*T*_, *u*_0:*T*_)). The costs *C*(*x*_0:*T*_, *u*_0:*T*_) play the role of the neg-log-probability of *w_t_* = 1, in accordance to the typical identification of energy with neg-log-probability The distance to obstacles is treated as a separate task and it enters the model through a task variable *y_t_* that represents position in the task space (see Figure 4). Readers are refered to [16,30-33] for more details how to couple the task variables with states. We compute the posterior *P*(*x*_0:*T*_∣*w*_0:*T*_ = 1) over the state trajectories to solve the path planning problem using the Gaussian message passing algorithm. This involves combining the forward (*μ*_*x*_*t*−1___→*x_t_*_), backward ((*x_t_*)*μ*_*x*_*t*+1_→*x*_*t*__) and cost messages ((*x_t_*)*μ*_*w*_*t*_→*x*_*t*__(*x_t_*)) to compute the posterior marginal belief:

(7) $$b(x_{t})=\mu_{x_{t-1}\to x_{t}}(x_{t})\mu_{x_{t+1}\to x_{t}}(x_{t})\mu_{w_{t}\to x_{t}}(x_{t})$$

The cost function in AICO is defined by task variables. Each task variable defines a task space and a squared metric is used to compute the cost inferred in this space. However, inference-based path planning with the linearised motion model and the holonomic constraint is difficult and suffers from problems with local minima due to the velocity constraints. The reasoning behind this is that the Gaussian distribution over the state space can potentially assign probability mass to states that do not satisfy the holonomic constraint, which either causes sideways slipping in the model or if we constrain the Gaussian itself the distribution becomes degenerate. For this reason, we have excluded the orientation from the state and we have added an additional cost term to penalise for angular velocity instead. We assume that arbitrary angular velocities can be executed but optimise for low angular velocities. This reduces the complexity of the state space by turning the hard holonomic constraint into a soft constraint.

**Figure 5.**
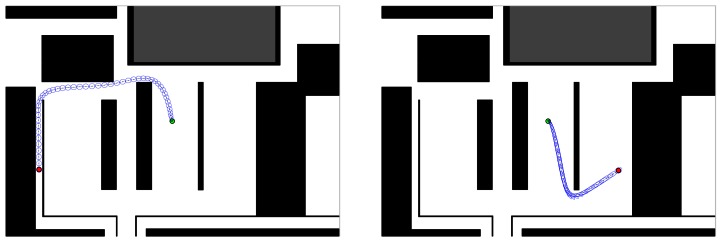
Two examples of optimal paths computed using AICO in a static environment.

In order to achieve robustness and safety of our vehicle, we use a method that yields free paths that tend to maximise the clearance between the vehicle and the obstacles based on a Voronoi graph. The aim of the global planning is to keep the vehicle at a safe distance from the surrounding obstacles. We compute the initial path using graph search on the Voronoi graph. This path is then used as AICO initialisation and it helps to deal with local minima. Figure 5 shows examples of optimised paths computed for static environments.

## 3. Implementation and Results

In this section we describe the implementation of our system and the experimental results. The results have been obtained from the real Seekur Jr. platform and the simulator provided by Mobilerobots.

The results have been evaluated in two stages: firstly we evaluate the gain of using the probabilistic model of the environment inside the perception stage of the classical algorithms (VFH+, CVM, LCM and BCM). Secondly, we compare our whole dynamic obstacle avoidance system with the classical algorithms.

### 3.1. Test-Bed

**Figure 6.**
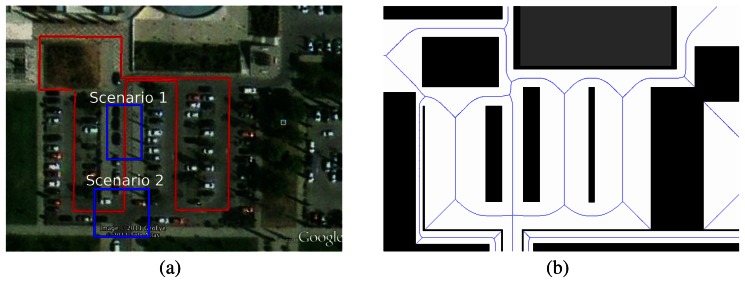
Test environment. (**a**) Surveying route and test scenarios in real environment; (**b**)Voronoi Diagram.

We have tested our system in an outdoor environment in the South Parking of the Polytechnic School at the University of Alcalá (UAH). The overall area of the environment is approximately 70 × 70 *m*^2^ (Figure 6(a)). In addition, the surveying route has been marked in red colour. The route was a 330 m long, and the blue rectangles represents the scenarios where the system was tested.

**Figure 7.**
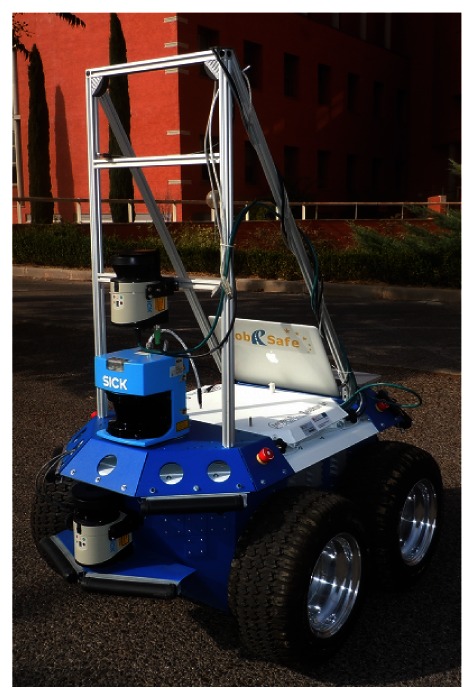
Seekur Jr. used in the experimentation.

The robot used in the experimentation was a Seekur Jr. (*c.f.*Figure 7), with the following configuration: MacBook Pro with Ubuntu 12.04 LTS operating system, Aria/MobileSim control software, RTK-GPS Maxor GGDT by JAVAD, low-cost GPS and stereo camera, two SICK LMS 151 outdoor lasers (the first one parallel to the ground and the other mounted at an angle to obtain the 3D points cloud), bumpers, encoders in the wheels and one Inertial Measurement Unit (IMU) to reduce the odometry errors in the turns.

### 3.2. Results Using the Probabilistic Model of the Dynamic Environment

We used four classical algorithms on the real platform and analysed the effect of using our proposed probabilistic model. For simplicity, we only take into account the *L*2 level of the three-dimensional BOF estimation (at the height of main body of the Seekur). We have tested the robot in two different scenarios and commanded it to reach a goal 7 meters away from its start position:

1. Parallel: For this test, the robot is located in the middle of a 5 meters wide corridor (Figure 6(a): Scenario 1). The obstacle starts moving along the corridor in the same direction as the robot but with a delay and it tries to overtake the robot.
2. Perpendicular: For this test the robot is located at the crossroads (Figure 6(a): Scenario 2). The obstacle is moving along the main road perpendicular to the robot's direction, blocking its path.

For all of these experiments, we have analysed the following parameters:

- Path curvature: The assumption is that the smoother the path, the lower the energy consumption.
- Acceleration (*a*): the positive acceleration in (*ms*^−2^) ignoring energy regenerated from breaking.
- Velocity (*v*): the absolute velocity of the robot in (*ms*^−1^).
- Time (*t*): the time needed to reach the goal in (*s*).
- Energy (*E*): the energy consumption of the robot in (*J*).

**Figure 8.**
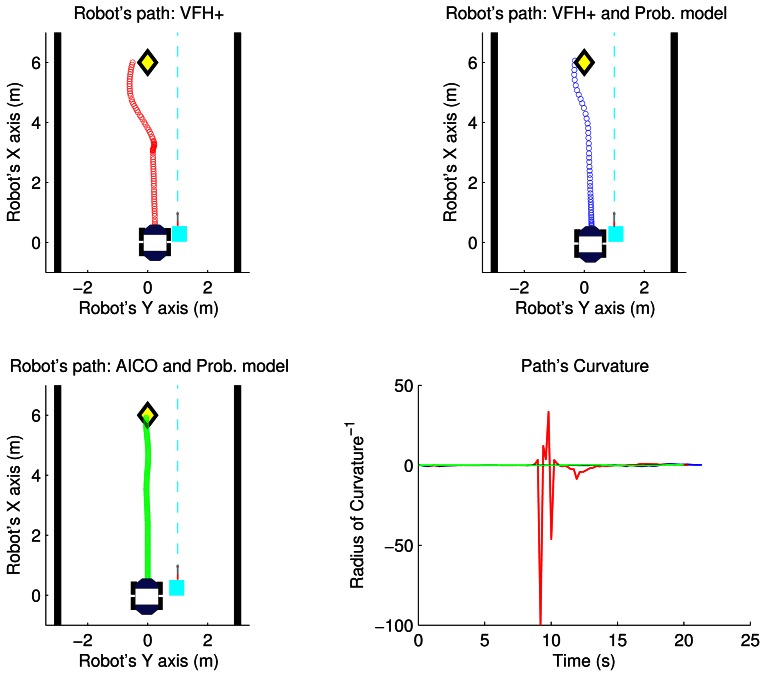
Path using VFH+ and AICO: parallel scenario.

**Figure 9.**
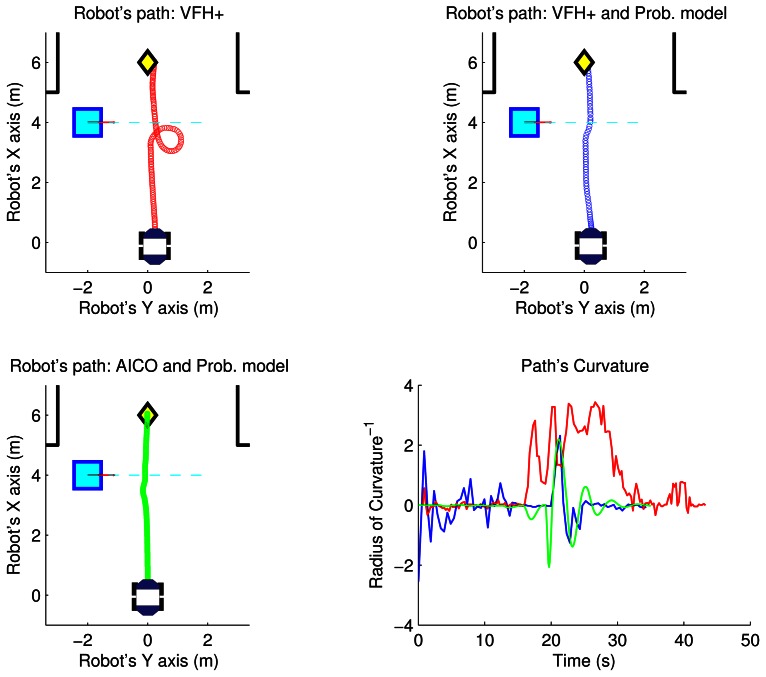
Path using VFH+ and AICO: perpendicular scenario.

As an example, the top part of the Figures 8 and 9 shows the path followed by the robot using the VFH+ algorithm and VFH+ with our probabilistic model in the two scenarios. The yellow diamond is the target goal. The plots show that all paths obtained by our proposed probabilistic method are smoother with lower overall curvature.

**Figure 10.**
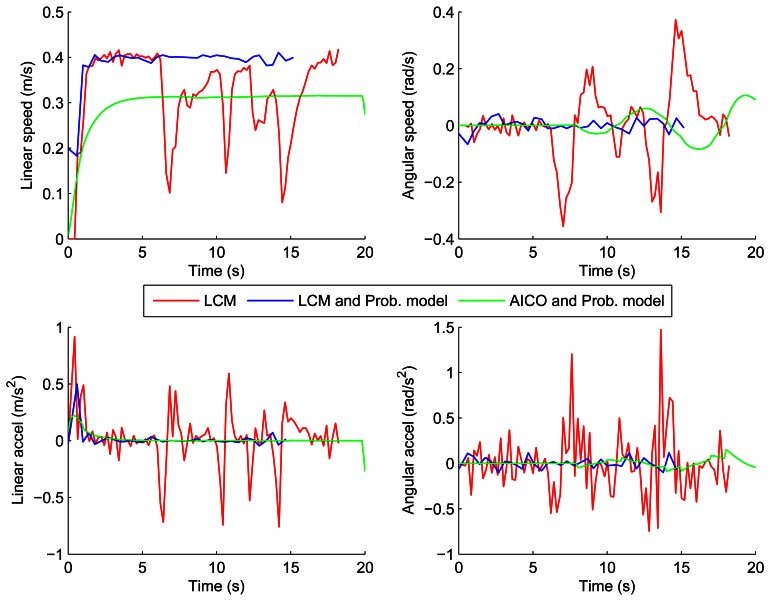
Velocities and accelerations using LCM and AICO: parallel scenario.

**Figure 11.**
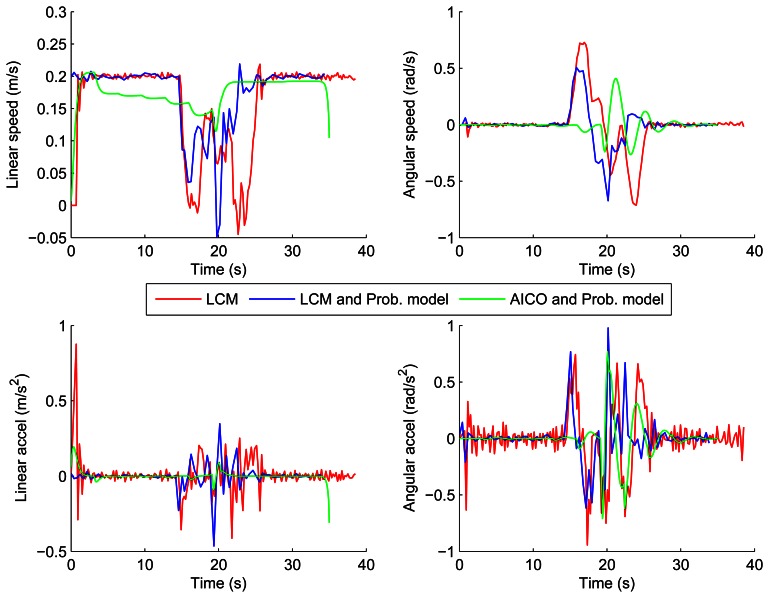
Velocities and accelerations using LCM and AICO: perpendicular scenario.

Figures 10 and 11 show the velocities and accelerations of the robot using the LCM and our probabilistic model in both scenarios. The results show that the accelerations and velocities over time are also much smoother and lower when using our probabilistic model of the environment.

**Figure 12.**
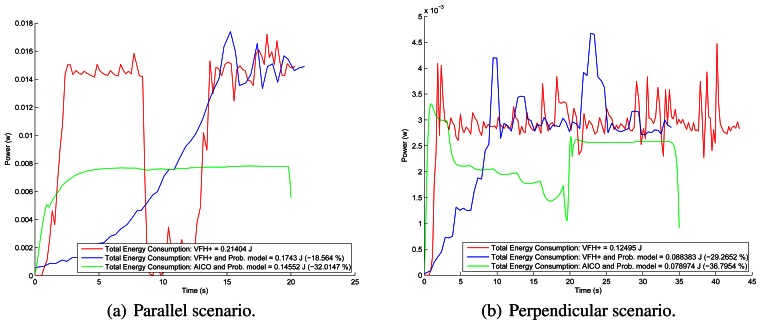
Energy consumption using VFH+ algorithm.

Figure 12 shows the energy consumption of the robot using the VFH+ algorithm in both scenarios. The results show that using our probabilistic model, the energy consumption is reduced by 20%. A summary of energy consumption is shown in Table 1. We can conclude that the use of our probabilistic model of the environment in classical algorithms reduces the energy consumption up to 45.5% in this setting.

### 3.3. Results of the Dynamic Obstacle Avoidance System

The aim of this experiment is to show that our system computes safe paths, reaching the goal configuration optimally with respect to energy consumption. Here we show that the performance of the reactive methods can be further improved by optimising the motion with respect to energy. The inference based planner described in Section 2.3 is initialized using the path computed from the Voronoi graph of the environment including only static obstacles. Then AICO computes the initial optimal path from start to goal positions (Figure 5). Starting and goal positions are marked by the green and red dots respectively. The covariance ellipses are overlaid.

We have used our probabilistic model of the environment to detect the position and velocity of dynamic obstacles in the robot coordinate frame which we have then mapped into the global coordinate frame. We use this information to predict the movement of these obstacles. AICO is then used to compute the optimal trajectory around the initial path while using the probabilistic model predictions about the dynamic obstacles. A set of task variables has been used to define the optimality: position, power demand (Equation (3)), turning velocity and collision avoidance. The collision avoidance is achieved by inferring cost for reciprocal distance to the closest obstacle. AICO works under the assumption that the full state of the world, including the motion of the obstacles is known. This is, however, no longer true when the prediction made by our probabilistic model is inaccurate. We therefore update our prediction using the new observations and discount the occupancy probability over time. We re-plan the path if the prediction error reaches a threshold. The planner therefore behaves similarly to a Kalman filter.

Only small changes in the environment between two time steps are expected. In such situations AICO requires only small number of iterations to converge which makes re-planning computationally affordable. The replanning time (3 s on average) is however too long to be use as a reactive controller.

We have applied our proposed method to perform the task while avoiding the obstacles and minimising energy consumption. AICO solves the finite horizon optimisation problems which means that the duration of the trajectory needs to be specified a priori. It is not within the scope of this paper to optimise for time, we have therefore set the trajectory duration to 20 s for parallel scenario and 35 s for perpendicular scenario, which are the respective average durations as computed using the reactive methods with our probabilistic model.

Figures 8 and 9 show the results of the path optimization and demonstrate that optimising the energy consumption further decreases the curvature of the trajectory. Similarly, Figures 10 and 11 show that the velocity and acceleration profiles are much smoother. As a result the energy consumption in both scenarios was reduced by approximately 10% when compared with the best results achieved by the reactive methods as shown in Table 1.

## 4. Conclusions and Future Works

In this paper, we have presented a method for avoiding dynamic obstacles, taking into account not only the integrity of the system, but also the minimization of the energy consumption. We have proposed a probabilistic model of the dynamic environment using: (1) an extension of the Bayesian Occupancy Filter proposed by [14] to a three-dimensional method to detect the obstacles positions and (2) a method to estimate the velocity of these obstacles using a tracking stage based on optical flow and a detection stage based on blob filtering.

We have used the probabilistic model of the environment inside the perception stage of the four classical methods: VFH+, BCM, CVM and LCM. Just by using a single level of the occupancy grid we were already able to improve the perception stage of these avoidance systems. The results have shown an improvement in energy consumption up to 45.5%. We have also shown that the resulting trajectories as well as the velocity and acceleration profiles are much smoother when using our method.

We have implemented a Voronoi graph based global path planner which serves as an initialisation method for our inference based local planner: AICO. Within AICO we combine multiple task variables to obtain the optimal path based on obstacle clearance and energy consumption. This method shows further qualitative improvement against the reactive methods with an improvement in energy consumption of 10.91% and 16.51% respect to the best results of reactive methods in perpendicular and parallel scenarios respectively. The computational cost of using AICO is currently too high for it to be used in real-time planning applications.

In near future, we intend to improve the accuracy of the probabilistic model by refining the discretisation. We also intend to improve the calculation of the probabilistic model to speed up the algorithm to accommodate for higher number of cells. The planning algorithm can be improved by solving the inference at multiple scales and by exploiting parallel computation. Furthermore, we will address issues of coverage using topology based properties such as winding numbers [34]. This will allow us to reduce energy consumption by planning optimal route to survey an area such as a parking lot when searching for a parking space.

## Figures and Tables

**Table 1.** Summary of results for energy consumption. AICO is being compared with the best reactive method for the given scenario.

	Scenario	Perception stage	Total Energy Consumption (J)	Reduction of Consumption (%)
VFH+	Parallel	Raw laser data	0.21404	–18.56%
		Probabilistic model	**0.1743**	
	Perpendicular	Raw laser data	0.12495	–29.26%
		Probabilistic model	0.088383	
CVM	Parallel	Raw laser data	0.22615	–7.52%
		Probabilistic model	0.20914	
	Perpendicular	Raw laser data	0.10458	–4.39%
		Probabilistic model	0.099981	
LCM	Parallel	Raw laser data	0.20125	+2.89%
		Probabilistic model	0.20709	
	Perpendicular	Raw laser data	0.09097	−8.1295%
		Probabilistic model	**0.083575**	
BCM	Parallel	Raw laser data	0.20546	–7.29%
		Probabilistic model	0.19047	
	Perpendicular	Raw laser data	0.16599	−45.5%
		Probabilistic model	0.090454	
AICO	Parallel	Probabilistic model	**0.14552**	−16.51%
	Perpendicular	Probabilistic model	**0.0789**	−10.91%
